# Predicting Poor Outcome Before Endovascular Treatment in Patients With Acute Ischemic Stroke

**DOI:** 10.3389/fneur.2020.580957

**Published:** 2020-10-15

**Authors:** Lucas A. Ramos, Manon Kappelhof, Hendrikus J. A. van Os, Vicky Chalos, Katinka Van Kranendonk, Nyika D. Kruyt, Yvo B. W. E. M. Roos, Aad van der Lugt, Wim H. van Zwam, Irene C. van der Schaaf, Aeilko H. Zwinderman, Gustav J. Strijkers, Marianne A. A. van Walderveen, Mariekke J. H. Wermer, Silvia D. Olabarriaga, Charles B. L. M. Majoie, Henk A. Marquering

**Affiliations:** ^1^Department of Biomedical Engineering and Physics, University of Amsterdam, Amsterdam, Netherlands; ^2^Department of Clinical Epidemiology and Biostatistics, University of Amsterdam, Amsterdam, Netherlands; ^3^Department of Radiology and Nuclear Medicine, University of Amsterdam, Amsterdam, Netherlands; ^4^Department of Neurology, Leiden University Medical Center, Leiden, Netherlands; ^5^Department of Neurology, Erasmus MC - University Medical Center, Rotterdam, Netherlands; ^6^Department of Public Health, Erasmus MC - University Medical Center, Rotterdam, Netherlands; ^7^Department of Radiology and Nuclear Medicine, Erasmus MC - University Medical Center, Rotterdam, Netherlands; ^8^Department of Neurology, Amsterdam UMC, University of Amsterdam, Amsterdam, Netherlands; ^9^Department of Radiology, Cardiovascular Research Institute Maastricht, Maastricht University Medical Center, Maastricht, Netherlands; ^10^Department of Radiology, University Medical Centre, Utrecht, Netherlands; ^11^Department of Radiology, Leiden University Medical Center, Leiden, Netherlands

**Keywords:** ischemic stroke, prediction modeling, machine learning, functional outcome, poor outcome, MRS, endovascular treatment (EVT)

## Abstract

**Background:** Although endovascular treatment (EVT) has greatly improved outcomes in acute ischemic stroke, still one third of patients die or remain severely disabled after stroke. If we could select patients with poor clinical outcome despite EVT, we could prevent futile treatment, avoid treatment complications, and further improve stroke care. We aimed to determine the accuracy of poor functional outcome prediction, defined as 90-day modified Rankin Scale (mRS) score ≥5, despite EVT treatment.

**Methods:** We included 1,526 patients from the MR CLEAN Registry, a prospective, observational, multicenter registry of ischemic stroke patients treated with EVT. We developed machine learning prediction models using all variables available at baseline before treatment. We optimized the models for both maximizing the area under the curve (AUC), reducing the number of false positives.

**Results:** From 1,526 patients included, 480 (31%) of patients showed poor outcome. The highest AUC was 0.81 for random forest. The highest area under the precision recall curve was 0.69 for the support vector machine. The highest achieved specificity was 95% with a sensitivity of 34% for neural networks, indicating that all models contained false positives in their predictions. From 921 mRS 0–4 patients, 27–61 (3–6%) were incorrectly classified as poor outcome. From 480 poor outcome patients in the registry, 99–163 (21–34%) were correctly identified by the models.

**Conclusions:** All prediction models showed a high AUC. The best-performing models correctly identified 34% of the poor outcome patients at a cost of misclassifying 4% of non-poor outcome patients. Further studies are necessary to determine whether these accuracies are reproducible before implementation in clinical practice.

## Introduction

Over the past 4 years, endovascular thrombectomy (EVT) unquestionably proved its value in anterior circulation acute ischemic stroke (1, 14–20). Despite the encouraging results, however, still ~30% of patients die or remain dependent of daily nursing care after EVT, making their treatment benefit essentially minimal (17, 18).

If we could reliably select patients with poor outcome after stroke despite EVT, we could spare patients a futile treatment with a needless risk of complications and enable a more efficient use of resources (21). Unfortunately, so far, no studies have been able to definitively identify a subgroup of patients that should not be treated with EVT (21).

In patient selection, it could be useful to predict poor outcome. Many previous studies focused on predicting functional independence after EVT (22). However, the use of such models would raise an ethical question. If a model predicts a zero percent chance of functional independence with EVT for a patient, one might advise to not treat. Untreated, the patient likely has a worse outcome, possibly needing continuous care in a nursing home. Treated, the patient may be able to function with some assistance in daily activities. Should we not treat this patient? A more valuable argument could be a reliable prediction of death or complete dependence of continuous care, even after EVT.

Some studies, such as MR PREDICTS, used data from randomized trials to predict treatment benefit as a modified Rankin Scale (mRS) score shift, using ordinal logistic regression (13). Predicting treatment benefit can be useful: if a patient is predicted to benefit from EVT in addition to regular care, one would proceed with EVT. However, data from randomized trials are necessary for such a model because predicted outcomes need to be based on a sufficient number of patients who did or did not receive EVT without indication bias. The amount of available data from randomized trials on EVT is limited. No new data after the HERMES trials will be available to train and validate models (17). An outcome measure that can enable long-term model improvement such as poor functional outcome could be of added value to models predicting treatment benefit.

Only a few studies have used poor outcome as their outcome measure; however, they had a limited amount of data and focused on linear classifiers (23). Machine learning (ML) may be of added value in predicting outcome after EVT. The number of relevant prognostic factors in stroke patients is high, and their effects on outcome may be indirect, combined, or otherwise complicated. With the ability to identify relevant prognostic variables through linear and non-linear relationships, ML may have added value in poor outcome prediction.

ML belongs to the artificial intelligence domain, where algorithms are designed to automatically learn patterns from data. In the work by Van Os et al. (22), ML methods predicted functional independence after acute ischemic stroke in a large population (1,383 patients), with reasonable certainty [area under the curve (AUC) 0.79].

Since the addition of EVT to standard care, the amount of available outcome data has greatly increased, now allowing for more powerful and elaborate prediction modeling. In the current study, we aim to assess the accuracy of pre-procedural prediction of poor functional outcome after EVT using ML models in patients from the MR CLEAN Registry.

## Methods

### Study Population

We included patients from the MR CLEAN Registry, which is a prospective, observational, multicenter study, consecutively including all EVT-treated acute ischemic stroke patients in the Netherlands since the completion of the MR CLEAN trial (24) in March 2014. The MR CLEAN Registry contains data from 16 centers distributed across The Netherlands. The current study is a retrospective report on patients included in the MR CLEAN Registry between March 2014 and June 2016 with intracranial proximal occlusions of the anterior arterial circulation (internal carotid artery (ICA) or internal carotid artery terminus (ICA-T), middle (M1/M2) or anterior (A1/A2) cerebral artery), aged ≥18 years, and treated in a MR CLEAN trial center. Patients were treated with intravenous thrombolysis (IVT) before EVT, if eligible. The central medical ethics committee of the Erasmus Medical Center Rotterdam, the Netherlands, evaluated the study protocol and granted permission (MEC-2014-235) to carry out the data collection as a registry (18). The procedures followed were in accordance with institutional guidelines. Patients provided permission for study participation through an opt-out procedure. The data can be made available on reasonable request from the MR CLEAN Registry committee (mrclean@erasmusmc.nl). All code used for the development of the models and data analysis is available at https://github.com/L-Ramos/MrClean_Poor. All imaging was assessed by an independent core laboratory, composed of 21 observers (20 interventional neuro- and/or interventional radiologists and one interventional neurologist) who were blinded to all clinical findings, except for symptom side. Assessed baseline imaging modalities were non-contrast CT [dense vessel sign, Alberta Stroke Programme Early CT Score (ASPECTS), hemorrhage, old infarcts, leukoaraiosis], CT angiography (CTA; occlusion location, clot burden score, collateral grade), and digital subtraction angiography (DSA; successful reperfusion, defined as extended thrombolysis in infarction score 2B-3). Other imaging variables that have proven to be predictive for outcome such as stroke lesion shape and size are difficult to observe on CT scans and were therefore not included in our models (25).

### Study Variables, Outcome, and Missing Data

Provided the correct methodology is used, ML methods allow the analysis of a large number of features. Therefore, we analyzed all 51 patient variables collected at baseline before treatment. Ordinal variables such as pre-stroke mRS, collaterals, ASPECTS, National Institutes of Health stroke scale (NIHSS), clot burden score, and Glasgow Coma Scale were treated as linear continuous scores. Some variables like time to groin puncture, despite not being readily available at baseline, can be estimated. If groin puncture is estimated to be possible within 6 h, patients can be treated within the regular EVT time window. In addition, achievable door-groin time of <60 min is currently used as inclusion criterion for several acute stroke trials (such as MR CLEAN-NO IV; ISRCTN80619088). More details about the included variables, distributions, and how they were included in the models are listed in Supplementary Table 1.

The outcome measure of interest of this study was poor functional outcome, defined as an mRS score of ≥5 at 90 days after stroke. Data on the mRS were collected by the MR CLEAN Registry hospitals as part of usual care (18).

Missing baseline and outcome data [mRS, *n* = 125 (8%)] were imputed using two approaches: a multiple imputation approach using Multiple Imputation by Chained Equations (MICE) (11), which is the most commonly used in literature (and the standard for MR CLEAN Registry–based studies) and a single imputation approach using Random-Forest Imputation (RFI) (26), which is a more recent, state-of-the-art imputation method. Variables with more than 40% missing were excluded from the analysis.

### Machine Learning Methods

We applied the following ML methods:

- Random forest classifier (RFC) (27), an ensemble classifier that combines many decision trees trained individually. Each decision tree is trained on random samples from the dataset, which reduces the variance of the prediction without increasing the bias;
- Support vector machine (SVM) (28), which separates classes by constructing hyperplanes and maximizing the margin in a multidimensional space;
- Artificial neural networks (NN) (6), which is composed of many interconnected nodes arranged in layers, where information is propagated from the first input layer up to a final output layer that delivers a prediction;
- Gradient boosting (XGB) (7), which is also an ensemble classifier that uses decision trees, but instead of training the trees individually, Gradient Boosting trains the trees sequentially, gradually improving them based on the previous ones; and
- Logistic regression (LR), which models the probability of a binary outcome using a linear function of the predictor variables.

Because there are many ML methods described in the literature, for which learning occurs in very different ways, we selected models that differ in learning procedure to increase the chance of developing models that generalize well (2). These methods have shown state-of-the-art results in several stroke-related applications (3, 22, 29). For the Gradient Boosting method, we used the implementation from https://github.com/dmlc/xgboost (7). For all the other methods, we used the implementations from *Scikit Learn* toolkit version 0.21.3 (4).

### Machine Learning Pipeline

We used a nested cross-validation (CV) strategy for model optimization and evaluation. In the outer CV loop, the dataset was split into 10 equally sized folds. For each CV iteration, 9-fold were used as training set and one was used as test set. In the inner CV loop, the training set was again divided into 5-fold (four used for training and one for validation), used for training the RFI imputer and determining the best hyper-parameters for all ML models. Hyper-parameters are parameters specific to each ML method. Their values cannot be automatically learned by the methods. The hyper-parameters were optimized using the random grid search function available on *Scikit Learn* (4), for maximizing the AUC. A list of the hyper-parameters used can be found in Supplementary Table 2, together with a description of the optimization procedure and choice of values.

For the LR models, we used feature selection using LASSO to define a subset of relevant variables. Creating a subset avoids diluting the coefficients of the model, which can form a challenge in interpreting variable importance (8).

Because the outcome variable was slightly imbalanced, and class imbalance can bias some classifiers, we applied balanced class weights during training of all models (4, 30). Class weights change the way the loss is calculated. The individual errors are multiplied by a sample weight, which shifts the minimum of the loss function. This way, when the error is high for a sample from a less prominent class, its impact will be higher in the loss, leading to a larger penalization in the whole model. We chose this approach because it has shown to work well even when class imbalanced in severe (up to thousands of times fewer samples from a given class) (30).

### Model Performance

Model performance was evaluated on the testing sets. We evaluated model performance using AUC, sensitivity (poor outcome patients correctly classified as poor outcome), specificity (percentage of non-poor outcome patients correctly classified as non-poor outcome), positive predictive value (PPV) (predicted poor outcome patients actually having poor outcome), negative predictive value (NPV) (predicted non-poor outcome patients actually having non-poor outcome), Matthews Correlation Coefficient (MCC) (correlation coefficient between the observed and predicted classes that is robust to class imbalance) (31), and the area under the precision recall curve (AUPRC). A high AUPRC relates to high precision (low false positive rate) and recall (low false negative rate), and is also a robust measure for class imbalance (4).

We built 10 models for each ML method through cross-validation. Therefore, the measures were averaged over all iterations and 95% confidence intervals (CIs) were computed. To limit the number of false positives (and, consequently, the risk of withholding treatment from patients who may still have good functional outcome), we optimized the predictions from the models (probability of poor outcome) to maximize specificity; above or equal to 0.95, 0.98, and 1.00, using the validation dataset to determine a threshold for the probabilities. This threshold was determined based on incremental search, by continuously increasing the threshold in 0.01 units until specificity was equal or higher than 0.95.

To assess model performance, we used Grotta bars to visualize the mRS distribution of patients that were classified by the models into poor outcome vs. non-poor outcome. Per ML method, three Grotta bars were computed for a specificity threshold of 0.95, 0.98, and 1.00, to assess the impact of reducing the number of false-positive predictions. Finally, we investigated the variables with the most predictive value for the best-performing models (high PPV and small number of FP) using odds ratio for LR and permutation feature importance (32). In permutation feature importance, each variable is individually shuffled before training and the decrease in accuracy (or in our case, AUC) is computed. The more the AUC decreases, the more important the variable is for the model.

## Results

### Study Population

A total of 1,526 patients were included (Supplementary Figure 1). Mean age was 71 years, and median baseline NIHSS was 14 (Table 1). Successful reperfusion was achieved in 863/1,505 patients (57%), and 753/1,092 (69%) of patients with complete post-EVT DSA runs available. At 90 days, 480 (31%) patients had a poor functional outcome (mRS 5–6), whereas 921 (61%) did not [outcome missing in *n* = 125 (8%)].

**Table 1:** Baseline characteristics; overall compared with mRS 5–6 vs. 0–4.

**Characteristics**	**Total study sample *N* = 1,526**	**mRS 5–6 *N* = 480**	**mRS 0–4 *N* = 921**
Age (years)—median (IQR)	71 (60–79)	77 (69–84)	67 (55–75)
Male sex—*n* (%)	809 (53.0)	245 (51.0)	502 (54.5)
Diabetes—*n* (%)	145 (13.9)	117 (24.4)	262 (17.2)
Pre-stroke mRS—*n* (%) 0–2	1,327 (86.9)	370 (77.1)	957 (91.5)
3–5	172 (11.3)	95 (19.8)	77 (7.4)
NIHSS at baseline—median (IQR)	14 (9–18)	16 (12–20)	13 (8–16)
Systolic blood pressure (mmHg)—mean (SD)	150 (24.6)	154 (25.8)	147 (23.7)
Glucose level before EVT median (IQR)	6.7 (8.0–5.9)	7.2 (8.8–6.1)	6.6 (7.8–5.8)
Intravenous alteplase—n (%)	1,170 (76.7)	327 (68.1)	743 (80.7)
Onset to groin puncture time (min)—median (IQR)	210 (160–270)	219 (170–273)	200 (155–266)
Hyperdense artery sign—*n* (%)	773 (50.7)	248 (51.7)	459 (49.8)
ASPECTS subgroups—*n* (%) 0–4	95 (6.2)	39 (8.13)	51 (5.5)
5–7	351 (23.0)	120 (25.0)	198 (21.5)
8–10	1,013 (66.4)	292 (60.8)	639 (69.4)
Occlusion location—*n* (%) ICA-T	322 (21.1)	128 (26.7)	194 (18.6)
M1	842 (55.2)	242 (50.4)	600 (57.4)
M2	181 (11.9)	52 (10.8)	129 (12.3)
Intracranial ICA	85 (5.6)	21 (4.4)	64 (6.2)
Other (M3 or anterior)	19 (1.3)	6 (1.3)	13 (1.2)
Clot Burden Score—median (IQR)	6 (4–8)	6 (4–8)	6 (4–8)
Collateral score—*n* (%) 0	98 (6.4)	57 (11.9)	35 (3.8)
1	467 (30.6)	188 (39.2)	246 (26.7)
2	547 (35.8)	135 (28.1)	361 (39.2)
3	305 (20.0)	61 (12.7)	218 (23.7)

*EVT, endovascular treatment; ICA, internal carotid artery; IQR, interquartile range; mRS, modified Rankin Scale; NIHSS, National Institutes of Health stroke scale; ASPECTS, Alberta Stroke Programme Early CT Score*.

### Prediction Accuracy

For all models trained, the best average AUC was 0.81 (Table 2) for NN, and the best AUPRC was 0.69 for the SVM. In the test sets, the highest PPV was 0.69 for the NN and the highest NPV was 0.87 for SVM: from all non-poor outcome predictions, 79% of the patients indeed had a non-poor outcome. All models but the SVM showed higher values of specificity than sensitivity, with 0.89 being the highest specificity (for the NN). The NN showed also the highest MCC (0.45) and LR the highest balanced accuracy (0.73) (Supplementary Table 3).

**Table 2:** Evaluation measures in validation data for all poor outcome prediction models, trained to maximize the AUC.

**Method**	**Specificity**	**Sensitivity**	**PPV**	**NPV**	**AUC**	**AUPRC**
RFC	0.84 (0.81–0.86)	0.56 (0.51–0.62)	0.62 (0.56–0.68)	0.80 (0.78–0.83)	0.80 (0.77–0.82)	0.66 (0.61–0.72)
SVM	0.67 (0.61–0.72)	0.78 (0.75–0.81)	0.53 (0.48–0.57)	0.87 (0.84–0.89)	0.77 (0.74–0.76)	0.69 (0.65–0.74)
NN	0.89 (0.87–0.90)	0.53 (0.49–0.57)	0.69 (0.65–0.74)	0.80 (0.78–0.83)	0.81 (0.79–0.83)	0.68 (0.64–0.73)
XGB	0.79 (0.76–0.83)	0.63 (0.60–0.67)	0.59 (0.54–0.65)	0.82 (0.80–0.84)	0.78 (0.76–0.81)	0.64 (0.59–0.69)
LR	0.75 (0.73–0.78)	0.71 (0.68–0.73)	0.57 (0.53–0.62)	0.85 (0.83–0.86)	0.80 (0.78–0.82)	0.68 (0.63–0.74)

*The average of 10 cross-validation iterations is presented. RFC, random forest classifier; SVM, support vector machine; LR, logistic regression; XGB, gradient boosting; NN, neural networks; AUC, area under the curve; AUPRC, area under the precision recall curve; NPV, negative predictive value; PPV, positive predictive value*.

In Supplementary Table 4, the results for the probability threshold of 95% specificity are shown. Note that because the specificity is based on the training set, the actual specificity in the validation set is somewhat lower than 0.95. Because the probability thresholds were optimized for high specificity, values for sensitivity were low (highest 0.34 for NN), indicating a relatively high number of false negatives (poor outcome patients classified as non-poor).

NN was considered the most accurate model because it showed the highest PPV values (Table 2). For the probability threshold of 95% specificity, NN, XGB, and LR showed the best PPV results, and NN and LR showed the highest AUPRC results (Supplementary Table 4). They also had the highest NPV values among the other models. We did not find any difference between single imputation using Random Forest and multiple imputation using MICE; therefore, we used Random Forest imputation as default. The results for the MICE imputation approach are shown in Supplementary Table 5.

### Model Performance

**Figure 1:**
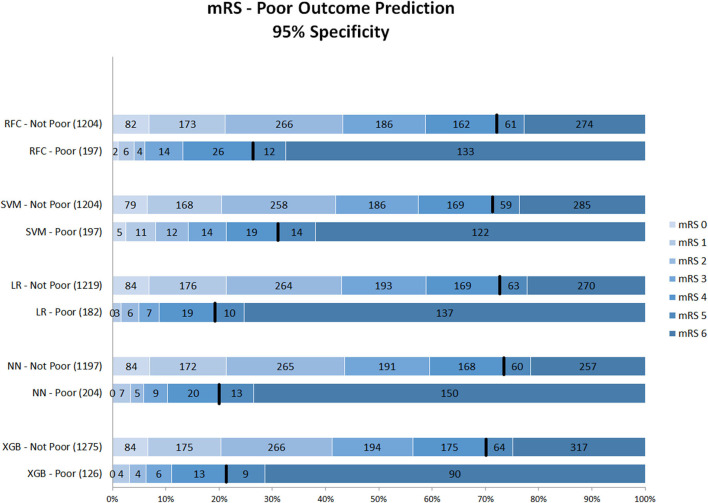
Distribution of mRS for the predictions of each model as poor vs. non-poor outcome with 95% specificity threshold. Along the y-axis, the various ML methods are presented including the number of patients who were classified as poor and non-poor outcome. Along the x-axis, the percentage of patients per mRS value is presented. In each graph, the black bar separates mRS 0–4 from 5 to 6. RFC, random forest classifier; SVM, support vector machine; LR, logistic regression; NN, neural network; XGB, gradient boosting; mRS, modified Rankin Scale. Numbers in bars represent absolute number of patients.

**Figure 2:**
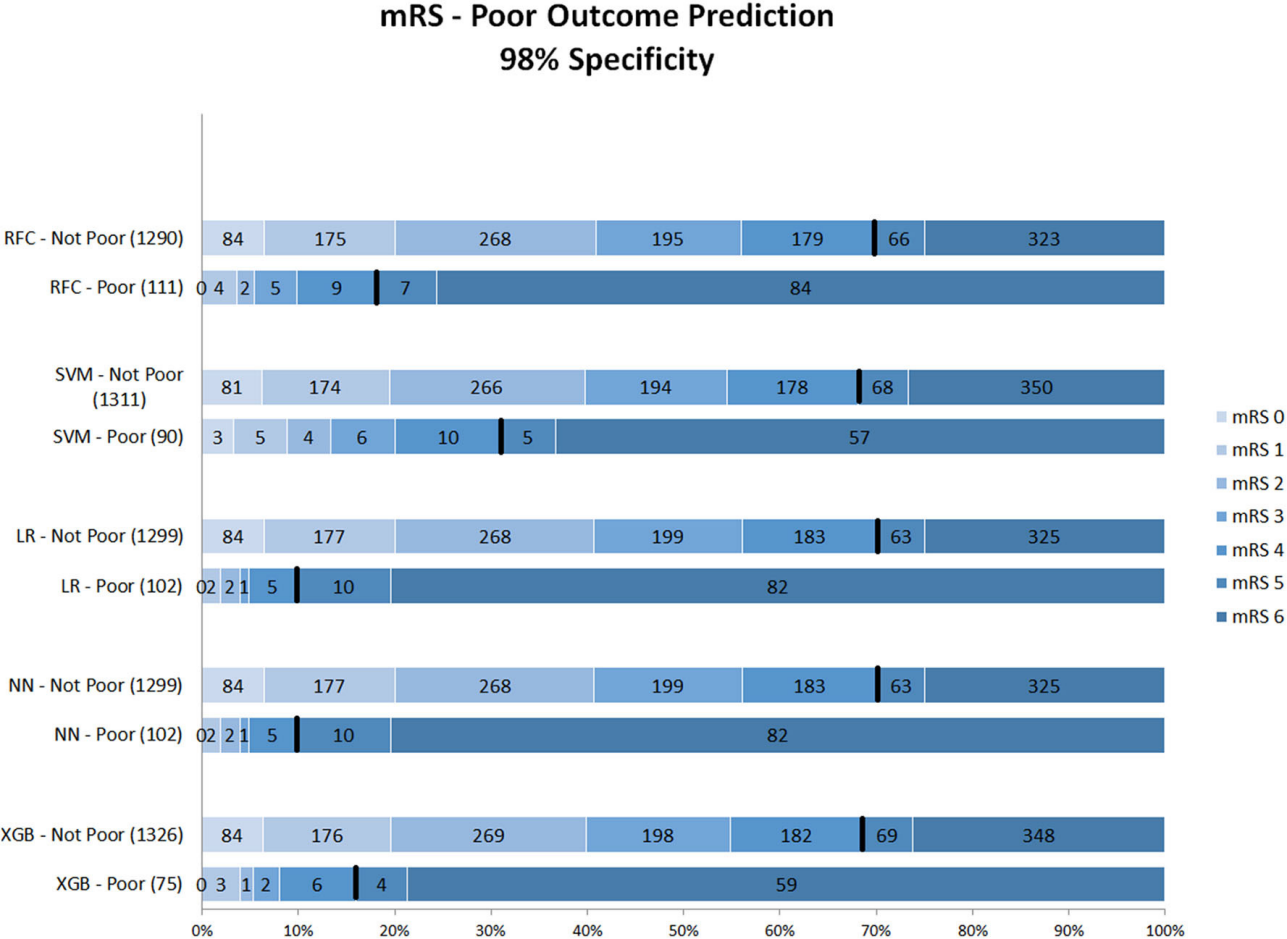
Distribution of mRS for the predictions of each model as poor vs. non-poor outcome with 98% specificity threshold. Along the y-axis, the various ML methods are presented including the number of patients who were classified as poor and non-poor outcome. Along the x-axis, the percentage of patients per mRS value is presented. In each graph, the black bar separates mRS 0–4 from 5 to 6. RFC, random forest classifier; SVM, support vector machine; LR, logistic regression; mRS, modified Rankin Scale; NN, neural network; XGB, gradient boosting. Numbers in bars represent absolute number of patients.

**Figure 3:**
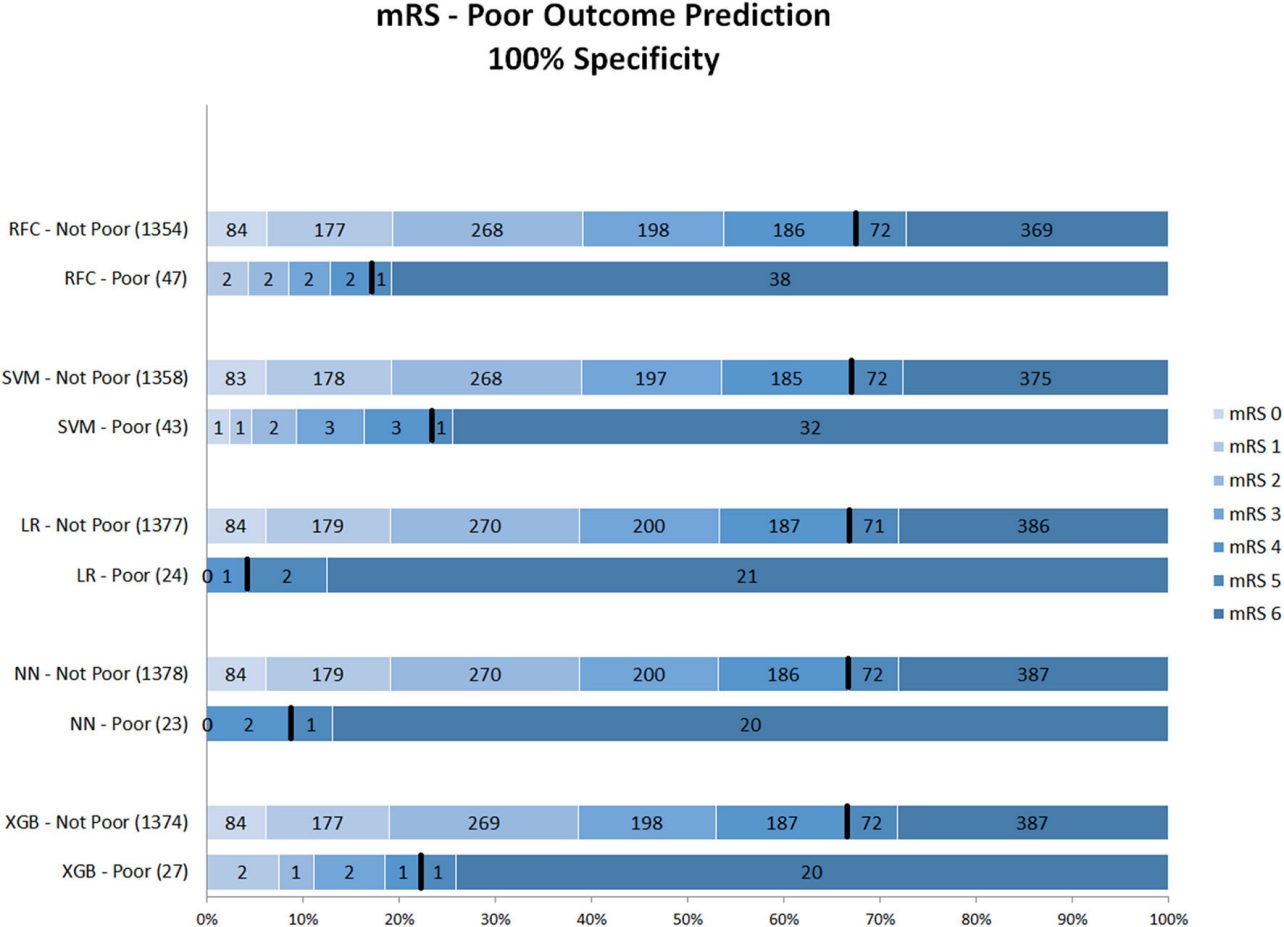
Distribution of mRS for the predictions of each model as poor vs. non-poor outcome with 100% specificity threshold. Along the y-axis, the various ML methods are presented including the number of patients who were classified as poor and non-poor outcome. Along the x-axis, the percentage of patients per mRS value is presented. In each graph, the black bar separates mRS 0–4 from 5 to 6. RFC, random forest classifier; SVM, support vector machine; LR, logistic regression; NN, neural network; XGB, gradient boosting; mRS, modified Ranking Scale. Numbers in bars represent absolute number of patients.

Figures 1–**3** show the mRS distribution outcome of patients classified as poor outcome and non-poor outcome in the testing data, for different specificity thresholds. For each ML method on the y-axis, we show how many patients were classified as poor and as non-poor outcome along the y-axis. Along the x-axis, the percentage of patients per mRS value is presented. In each graph, the black bar separates mRS 0–4 (non-poor outcome) from 5 to 6 (poor outcome). In Figure 1, the probability threshold was optimized to reach 95% specificity, and for some classifiers the rate of correct poor outcome prediction was higher than 80%. This is the case for LR, where from all poor outcome predictions, the total of mRS 0–4 patients is lower than 20%. However, all models still mistakenly predicted some mRS 0–4 patients as poor outcome (27 patients for the best model; <3% of all mRS 0–4 patients; Table 3). More patients were classified as non-poor outcome than poor outcome. For the NN model for example (Supplementary Figure 2), 11% (163/1,526) of all patients were classified as poor outcome, whereas 31% actually had a poor outcome.

**Table 3:** Number of false positives (mRS 0–4 classified as poor) and true positives (mRS 5–6 classified as poor) per specificity threshold for each ML method.

**Method**	**Optimized specificity**	**True positives mRS 5–6 patients classified as poor of total mRS 5–6 patients (*n* = 480)**	**False positives mRS 0–4 patients classified as poor of total mRS 0–4 patients (*n* = 921)**
RFC	95%	145 (30.2%)	52 (5.6%)
	98%	91 (19.0%)	20 (2.2%)
	100%	39 (8.1%)	8 (0.9%)
SVM	95%	136 (28.3%)	61 (6.2%)
	98%	62 (12.9%)	28 (3.0%)
	100%	33 (6.9%)	10 (1.1%)
NN	95%	163 (34.0%)	41 (4.5%)
	98%	92 (19.2%)	10 (1.1%)
	100%	21 (4.4%)	2 (0.2%)
XGB	95%	99 (20.6%)	27 (2.8%)
	98%	63 (13.1%)	12 (1.3%)
	100%	21 (4.4%)	6 (0.7%)
LR	95%	147 (30.6%)	35 (3.8%)
	98%	92 (19.2%)	10 (1.1%)
	100%	23 (4.8%)	1 (0.1%)

*RFC, random forest classifier; SVM, support vector machine; LR, logistic regression; NN, neural network; XGB, gradient boosting; mRS, modified Rankin Scale*.

Figure 2 shows mRS distributions for the probability threshold optimized to 98% specificity. The numbers of both correct and incorrect poor outcome predictions were reduced compared with the 95% threshold. Ten (1.1%) of mRS 0–4 patients were still misclassified as poor in the best-performing models (NN and LR); 92 poor outcome patients were correctly classified (Supplementary Figure 3).

Figure 3 shows the mRS distribution of patients that were classified as poor outcome vs. non-poor outcome in the validation data, for the probability threshold optimized to reach 100% specificity. Again, both correct and incorrect poor outcome predictions were reduced compared with the 95 and 98% thresholds. One (0.1%) patient was misclassified as poor outcome by LR and two (0.2%) by NN (Supplementary Figure 4). However, the ability to correctly identify poor outcome patients was reduced with 8.1% (*n* = 39) of poor outcome patients being correctly identified (RFC).

Table 4 shows the odds ratios for each variable included in the LR model. Baseline NIHSS, glucose level before EVT, age, 50% or more atherosclerotic stenosis at symptomatic carotid bifurcation on CTA, pre-stroke mRS, collateral score, leukoaraiosis, atrial fibrillation, and Glasgow coma scale were significantly associated with poor outcome.

**Table 4:** Odds ratio of each variable included in the logistic regression model.

**Variable**	**Odds ratio (95% CI)**
Age (years)	1.05 (1.04–1.06)
Pre-stroke mRS	1.35 (1.21–1.50)
Atrial fibrillation	1.37 (1.01–1.85)
NIHSS at baseline	1.06 (1.03–1.09)
Glucose level	1.16 (1.10–1.22)
Glasgow Coma Scale	0.90 (0.84–0.97)
Time: onset to groin puncture	1.00 (1.00–1.01)
50% or more atherosclerotic stenosis at symptomatic carotid bifurcation on CTA	0.61 (0.38–0.99)
ASPECTS on baseline NCCT	0.94 (0.88–1.01)
Leukoaraiosis	1.69 (1.28–2.24)
Collaterals	0.60 (0.51–0.70)

*CI, confidence interval; CTA, CT angiography; mRS, modified Rankin Scale; NIHSS, National Institutes of Health stroke scale; NCCT, non-contrast CT; ASPECTS, Alberta Stroke Programme Early CT Score*.

**Figure 4:**
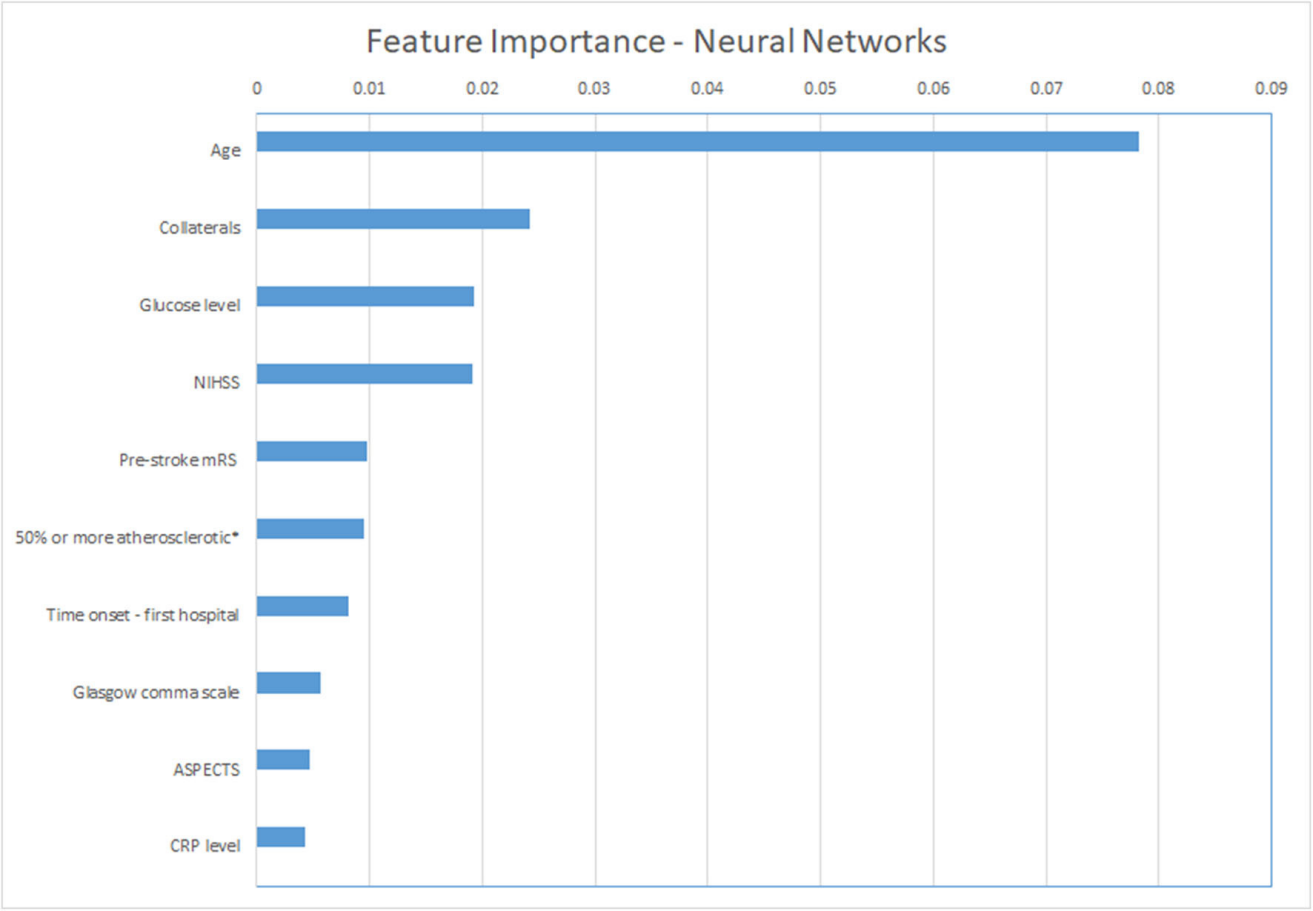
Permutation feature importance for the Neural Network models. Average impact on the AUC. *50% or more atherosclerotic stenosis at symptomatic carotid bifurcation on CTA baseline. ASPECTS, Alberta Stroke Programme Early CT Score; CRP, C reactive protein; mRS, modified Rankin Scale; NIHSS, National Institutes of Health stroke scale.

**Figure 5:**
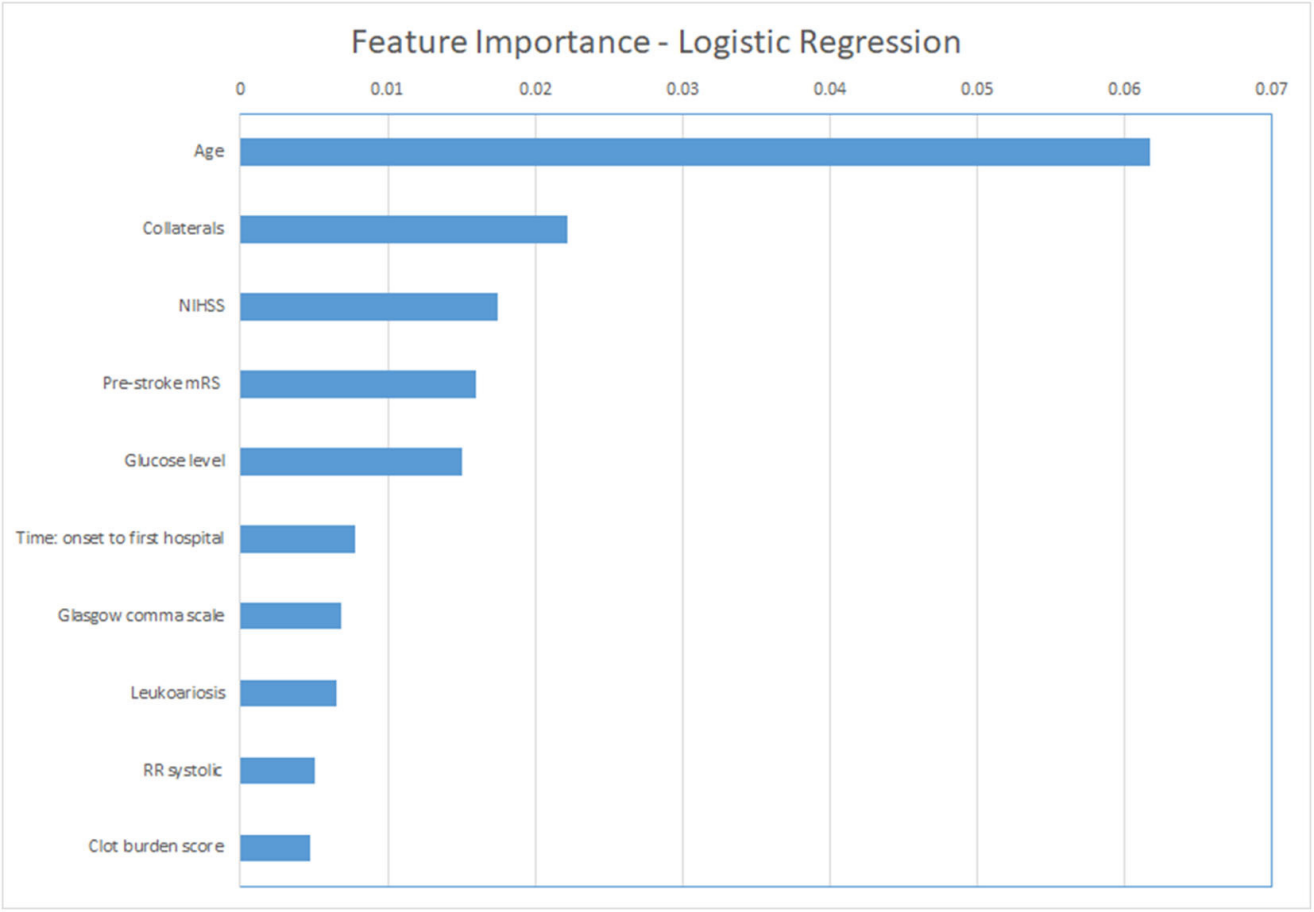
Permutation feature importance for the Logistic Regression models. Average impact on the AUC. mRS, modified Rankin Scale; NIHSS, National Institutes of Health stroke scale; RR, blood pressure (Riva-Rocci).

For the ML models, we show the permutation feature importance for the models with the least number of FP (LR and NN—Table 3) in Figures 4, 5. Permutation feature importance for the remaining ML methods is shown in Supplementary Figures 5–7. Age consistently shows the highest impact on the average AUC in all ML models. For both LR and NN, age, collaterals, glucose level, NIHSS, and pre-stroke mRS are ranked in the top 5 of the most important variables. In addition, RR diastolic at baseline and time from onset to first hospital were important variables for other ML models.

## Discussion

We have shown that poor outcome for acute ischemic stroke patients who were treated with EVT from the MR CLEAN Registry can be predicted with a high specificity. Although the models were optimized for high AUC and the thresholds optimized to high specificity, all models still classified some non-poor outcome patients as poor outcome, suggesting that these models are not yet accurate enough to be included in clinical practice.

To our knowledge, this is the first study to use multiple ML models and a large dataset for the prediction of poor functional outcome in acute ischemic stroke patients. Besides, our study included a larger number of variables than most stroke prediction models to date, so our study can be considered quite extensive (13). The presented accuracy was similar to the results of studies focusing on good functional outcome prediction, although a different cut-off for dichotomization could have impacted prediction accuracy and relevance of variables (13, 22).

All models showed similar performance in terms of AUC and AUPROC, although NN was the method with the highest PPV and specificity and was deemed the best-performing model in multiple experiments. ML models can also be compared in terms of complexity (training time, number of hyper-parameters, and interpretability) (33). However, this was beyond the scope of this study. Regarding the number of hyper-parameters, training time, and interpretability, LR is the best method, being simpler to handle while showing accuracies similar to the other more complex models. In Fatima and Pasha (33), it was shown that ML methods can greatly outperform each other in different datasets, although this was not the case in our study.

The most important features in our models were age (in all models), collaterals, glucose level, baseline NIHSS, onset-to-first hospital time, and pre-stroke mRS (in LR and NN, the models with the lowest false-positive rates). Recent studies that used the MR CLEAN Registry dataset found similar variables with the highest relevance for functional outcome using logistic regression (mRS ≤ 2): age, NIHSS, diabetes, and time from stroke onset to treatment (21, 22, 24). Interestingly, these studies also identified ASPECTS, location of occlusion, smoking, and hypertension as relevant, which were less frequently marked as important in our study (21, 22, 24). This may be related to the different dichotomization of mRS we used. Alternatively, it may have to do with the manual selection of variables for these logistic regression models, as opposed to the selection of included variables by the ML methods we used. Despite none of the currently known prognostic factors being selective enough to base any EVT exclusion decision on, the more or less intuitive importance of age, baseline stroke severity, and workflow times for a patient's outcome is confirmed in both our data and the mentioned previous studies.

Regarding poor outcome prediction, some studies have identified groups of patients that show poor outcome after IVT regardless of reperfusion using diffusion-weighted MRI and CT perfusion, respectively (5, 34).

## Strengths and Limitations

Strengths of our study include the large sample size and heterogeneity (coming from multi-centers) of the data, which includes patients from all over the Netherlands. One of the possible downsides of a heterogeneous dataset is that the models could learn the differences between centers instead of focusing on the task at hand (predicting poor outcome). Nevertheless, we made sure that no variables related to the individual centers were included and shuffled the dataset to prevent pre-determined patient clusters. Despite this downside, the benefits of having a heterogeneous dataset outweigh this risk because we aim to develop models on data that are closer to the clinical practice setting.

Furthermore, we explored distinct state-of-the-art ML methods and optimized their hyper-parameters using an inner CV loop, while testing the optimized model on the test sets in the outer CV, which helps to prevent overoptimistic results, increasing stability and reliability. We did not separate a unique test set due to risk of, by change, separating a dataset with easier or harder samples. We used several evaluation measures that allow the models to be assessed from different points of view, highlighting their differences. Our results show that there is little difference in AUC values between models. By using other measures, such as PPV, differences in performance between models became clearer.

Some limitations to the current study should be noted. Even though we used imputation to account for missing data, a bias in the imputed values can never fully be excluded because the estimates are always based on the available data. No difference between imputation using Random Forest and imputation using MICE was found. This can be due to the fact that the disadvantages of single imputation are mostly relevant in small datasets (with <100 events), which is not the case in the MR CLEAN Registry (9). Despite MICE being a more common imputation approach, RFI imputation is often more efficient than MICE as shown in previous studies (35), and we therefore only present the results for this approach. The large number of variables included can also be a limitation because some of the variables are not readily available or easily assessed and its assessment may delay treatment decision. However, all variables included can be derived before treatment decision (either by local radiologists or automated tooling). Another limitation lies in the models' performance. The number of patients classified as poor outcome became very low when specificity was set to a very high value, and models still had false positives. Furthermore, we used class weights to deal with data imbalance because other approaches, such as under-sampling, would lead to a distribution that is not realistic when compared with the real-life scenario of acute ischemic stroke. Finally, we did not use test sets during cross-validation or imputation, preventing information leakage between datasets, which could lead to overoptimistic results.

Part of the goals of this study was to study to what extent the ratio of correctly and falsely classified patients was with ML models. In the prediction of poor outcome, high specificity and PPV are important to avoid withholding treatment from patients that may still have a non-poor outcome after EVT. The ML models investigated in the current study had relatively high AUC, PPV, and specificity, although not all patients were correctly classified, even with a specificity threshold of 100%.

The ML methods applied in this study highlighted the relevance of several baseline factors in the prediction of poor functional outcome. For future research datasets, inclusion of variables such as glucose level should be considered. In daily practice, knowledge of the relevance of these variables could support decision-making by clinicians when combined with other relevant factors such as time from symptom onset and the patient's or family's wishes. Although the prognostic models included many baseline characteristics, other data of prognostic relevance derived from CTP imaging were not included because these were not commonly available in the data from the MR CLEAN Registry. The inclusion of these parameters have the potential to improve prediction in future studies. Besides, the more extensive follow-up NIHSS could be used to define poor functional outcome in future studies. Furthermore, for future research, ML models could be created using the raw imaging data (CT or CT angiography or both) and combined with the models created in this study (10, 12, 36, 37). However, the large number of data points has to be taken into account when developing such approaches because imaging data is often of high dimensionality, and medical datasets have often a very limited number of samples.

Finally, we used poor outcome as our primary outcome. Poor functional outcome could be a valuable outcome measure for further studies because the certainty of death or severe disability even after EVT could, to our expectations, form a relatively solid, ethically justifiable ground to refrain from EVT. That way, rates of futile treatment could be lowered. Poor outcome prediction may be useful as an outcome measure as an addition to the prediction of EVT benefit (mRS shift) because it does not require data from randomized trials and can hence be used to train models on future new data.

## Conclusion

Poor outcome can be predicted with high specificity, although all of the prediction models incorrectly classified some patients as poor outcome. The percentage of misclassified non-poor outcome patients was low, whereas more than one third of the poor-outcome patients were correctly identified. However, lowering false-positive rates came at the cost of decreased sensitivity. It has to be studied further whether these accuracies are reproducible before implementation in clinical practice could be considered or could be improved further. Age, NIHSS, baseline glucose levels, pre-stroke mRS, and collaterals were consistently ranked as important variables in all prediction methods.

## Data Availability Statement

Patients provided permission for study participation through an opt-out procedure. The data can be made available on reasonable request from the MR CLEAN Registry committee (mrclean@erasmusmc.nl).

## Ethics Statement

The studies involving human participants were reviewed and approved by the central medical ethics committee of Erasmus Medical Centre Rotterdam, the Netherlands (MEC-2014–235). The patients/participants provided their written informed consent to participate in this study.

## Author Contributions

LR: lead author, study design, analysis and interpretation, critical revision manuscript for important intellectual content. MK, HO, VC, and KV: study design, analysis and interpretation, critical revision manuscript for important intellectual content. NK, YR, AL, WZ, IS, MAAW, MJHW, and CM: data acquisition, critical revision of manuscript for important intellectual content. AZ, GS, SO, and HM: supervisors of lead author, study design, and critical revision of manuscript for important intellectual content. All authors contributed to the article and approved the submitted version.

## Conflict of Interest

Erasmus MC received funds from Stryker by AL. Amsterdam UMC received funds from Stryker for consultations by CM and YR. MUMC received funds from Stryker and Codman for consultations by WZ. CM reports grants from the TWIN Foundation, the CVON/Dutch Heart Foundation, the European Commission. HM is cofounder and shareholder of Nico.lab. CM and YR own stock in Nico.lab. The remaining authors declare that the research was conducted in the absence of any commercial or financial relationships that could be construed as a potential conflict of interest.
