# Supplementary material for: Predicting Poor Outcome Before Endovascular Treatment in Patients With Acute Ischemic Stroke
Source: Front Neurol. 2020 Oct 15;11:580957. doi: 10.3389/fneur.2020.580957 (PMC7593486; doi:10.3389/fneur.2020.580957)
Supplement: Supplementary file 1 [file Table_1.docx]

Supplementary Material

**Supplementary Table I. Details of included variables.** A1, first segment of anterior cerebral artery; ASPECTS, Alberta stroke programme early CT score; cat, categorical; CBS, clot burden score; cont, continuous; CRP, C-reactive protein; CTA, CT angiography; DOAC, direct oral anticoagulant; ER, emergency room; HAS, hyperdense artery sign; IQR, interquartile range; M1/M2/M3, first/second/third segment of middle cerebral artery; mRS, modified Rankin Scale; NCCT, non-contrast CT; NIHSS, National Institutes of Health stroke scale; RR, blood pressure (Riva-Rocci).

| **Name** | **Occurrence (%)**  **N=1526** | **Missing**  n (%) | **Analyzed as** |
| --- | --- | --- | --- |
| Previous stroke |  | 9 (1) | cat |
| 0 – no | 1264 (83) |  |  |
| 1 – yes | 253 (17) |  |  |
| Myocardial infarction |  | 31 (2) | cat |
| 0 – no | 1262 (83) |  |  |
| 1 – yes | 233 (15) |  |  |
| Peripheral arterial disease |  | 30 (2) | cat |
| 0 – no | 1358 (89) |  |  |
| 1 – yes | 138 (9) |  |  |
| Diabetes |  | 9 (1) | cat |
| 0 – no | 1255 (82) |  |  |
| 1 – yes | 262 (17) |  |  |
| Hypertension |  | 19 (1) | cat |
| 1 – yes | 765 (50) |  |  |
| 0 – no | 742 (49) |  |  |
| Atrial fibrillation |  | 22 (1) | cat |
| 0 – no | 1169 (77) |  |  |
| 1 – yes | 335 (22) |  |  |
| Hypercholesterolemia |  | 49 (3) | cat |
| 0 – no | 1035 (68) |  |  |
| 1 – yes | 442 (29) |  |  |
| Antiplatelet use |  | 19 (1) | cat |
| 0 – no | 1001 (66) |  |  |
| 1 – yes | 506 (33) |  |  |
| DOAC use |  | 26 (2) | cat |
| 0 – no | 1463 (96) |  |  |
| 1 – yes | 37 (2) |  |  |
| Coumarin use |  | 11 (1) | cat |
| 0 – no | 1321 (87) |  |  |
| 1 – yes | 194 (13) |  |  |
| Heparin use |  | 19 (1) | cat |
| 0 – no | 1452 (95) |  |  |
| 1 – yes | 55 (4) |  |  |
| Blood pressure medication |  | 28 (2) | cat |
| 1 – yes | 781 (51) |  |  |
| 0 – no | 717 (47) |  |  |
| Statin use |  | 32 (2) | cat |
| 0 – no | 958 (63) |  |  |
| 1 – yes | 536 (35) |  |  |
| HAS on baseline NCCT |  | 87 (6) | cat |
| 1 – yes | 773 (51) |  |  |
| 0 – no | 666 (44) |  |  |
| Relevant (new) ischemia / hypodensity |  | 113 (7) | cat |
| 1 – yes | 928 (61) |  |  |
| 0 – no | 485 (32) |  |  |
| Hemorrhagic transformation |  | 95 (6) | cat |
| 0 – no | 1400 (92) |  |  |
| 1 – yes | 31 (2) |  |  |
| Leukoariosis |  | 87 (6) | cat |
| 0 – no | 941 (62) |  |  |
| 1 – yes | 498 (33) |  |  |
| Old infarcts in same ASPECTS region? |  | 76 (5) | cat |
| 0 – no | 1247 (82) |  |  |
| 1 – yes | 203 (13) |  |  |
| Intracranial atherosclerosis on CTA scored by core lab |  | 91 (6) | cat |
| 1 – yes | 853 (56) |  |  |
| 0 – no | 582 (38) |  |  |
| Sex |  |  | cat |
| Male | 809 (53) |  |  |
| Female | 717 (47) |  |  |
| Most proximal occlusion segment on CTA scored by core lab, based on CBS |  | 68 (4) | cat |
| Distal M1 | 471 (31) |  |  |
| Proximal M1 | 371 (24) |  |  |
| ICA-T | 322 (21) |  |  |
| M2 | 181 (12) |  |  |
| Intracranial ICA | 85 (6) |  |  |
| None | 13 (1) |  |  |
| M3 | 9 (1) |  |  |
| A2 | 3 (0) |  |  |
| A1 | 3 (0) |  |  |
| Smoking |  | 348 (23) | cat |
| 0 – no | 827 (54) |  |  |
| 1 – yes | 351 (23) |  |  |
| Inclusion on weekday or weekend |  |  | cat |
| 0 – weekday | 1133 (74) |  |  |
| 1 – weekend | 393 (26) |  |  |
| Admission between 17.00-08-00 (weekday)/ weekend or holiday. Based on ER time. |  |  | cat |
| 1 – office hours | 982 (64) |  |  |
| 0 – outside office hours | 544 (36) |  |  |
| Transfer from other hospital |  |  | cat |
| 1 – transfer | 822 (54) |  |  |
| 0 – no transfer | 704 (46) |  |  |
| Intravenous alteplase treatment |  | 3 (0) | cat |
| 1 – yes | 1170 (77) |  |  |
| 0 – no | 353 (23) |  |  |
| No abnormalities at symptomatic carotid bifurcation on CTA baseline by core |  | 264 (17) | cat |
| 0 – no abnormalities | 943 (62) |  |  |
| 1 – any abnormalities | 319 (21) |  |  |
| 50% or more atherosclerotic stenosis at symptomatic carotid bifurcation on CTA baseline |  | 264 (17) | cat |
| 0 – no | 1140 (75) |  |  |
| 1 – yes | 122 (8) |  |  |
| Atherosclerotic occlusion at symptomatic carotid bifurcation on CTA baseline by core lab |  | 264 (17) | cat |
| 0 – no | 1132 (74) |  |  |
| 1 – yes | 130 (9) |  |  |
| Floating thrombus at symptomatic carotid bifurcation on CTA baseline by core lab |  | 264 (17) | cat |
| 0 – no | 1241 (81) |  |  |
| 1 – yes | 21 (1) |  |  |
| Pseudo-occlusion at symptomatic carotid bifurcation on CTA baseline by core lab |  | 264 (17) | cat |
| 0 – no | 1180 (77) |  |  |
| 1 – yes | 82 (5) |  |  |
| Carotid dissection at symptomatic carotid bifurcation on CTA baseline by core lab |  | 264 (17) | cat |
| 0 – no | 1206 (79) |  |  |
| 1 – yes | 56 (4) |  |  |
| Occlusion side on CTA scored by core lab |  |  | cat |
| Left hemisphere | 820 (54) |  |  |
| Right hemisphere | 694 (45) |  |  |
| Neither | 12 (1) |  |  |
| In-hospital stroke |  | 525 (34) | cat |
| 0 – no | 857 (56) |  |  |
| 1 – yes | 144 (9) |  |  |
| Contraindications for IVT |  | 12 (1) | cat |
| 0 – no | 1178 (77) |  |  |
| 1 – yes | 336 (22) |  |  |
| Second occlusion in other territory present on CTA scored by core lab |  | 479 (31) | cat |
| 0 – no | 822 (54) |  |  |
| 1 – yes | 225 (15) |  |  |
| Collateral score on CTA scored by core lab |  | 109 (7) | cont |
| 100% of occluded area | 305 (20) |  |  |
| >50% but less <100% | 547 (36) |  |  |
| filling <50% of occluded area | 467 (31) |  |  |
| Absent collaterals | 98 (6) |  |  |
| Pre-stroke mRS |  | 27 (2) | cont |
| 0 | 1017 (67) |  |  |
| 1 | 195 (13) |  |  |
| 2 | 115 (8) |  |  |
| 3 | 98 (6) |  |  |
| 4 | 62 (4) |  |  |
| 5 | 12 (1) |  |  |
| 90-day mRS |  | 125 (8) | cat |
| 6 | 407 (27) |  |  |
| 2 | 270 (18) |  |  |
| 3 | 200 (13) |  |  |
| 4 | 188 (12) |  |  |
| 1 | 179 (12) |  |  |
| 0 | 84 (6) |  |  |
| 5 | 73 (5) |  |  |
| ASPECTS baseline scored by core lab – median (IQR) | 9 (7 - 10) | 67 (4) | cont |
| CBS at baseline – median (IQR) | 6 (4 - 8) | 255 (17) | cont |
| NIHSS at baseline – median (IQR) | 16 (11 - 20) | 30 (2) | cont |
| Glucose level at baseline – median (IQR) | 7 (6 - 8) | 173 (11) | cont |
| RR systolic at baseline – median (IQR) | 150 (131 - 165) | 43 (3) | cont |
| RR diastolic at baseline – median (IQR) | 80 (70 - 91) | 48 (3) | cont |
| INR at baseline – median (IQR) | 1 (1 - 1) | 276 (18) | cont |
| Thrombocyte count at baseline – median (IQR) | 236 (194 - 290) | 189 (12) | cont |
| CRP level at baseline – median (IQR) | 5 (2 - 11) | 307 (20) | cont |
| Age – median (IQR) | 71 (60 - 79) | 0 (0) | cont |
| Total glasgow coma scale at baseline – median (IQR) | 13 (11 - 15) | 45 (3) | cont |
| Duration from onset to groin in minutes – median (IQR) | 210 (160 - 270) | 0 (0) | cont |
| Duration: onset to IVT in minutes in first hospital – median (IQR) | 25 (19 - 33) | 677 (44) | cont |

Hyper-parameter optimization

In Supplementary Table II, we present the range of values used for hyper-parameter optimization. We strived to include the largest range of values possible for all hyper-parameters while keeping the search computationally efficient. For example, for the neural network architecture, we started with a small number of hidden layers with fewer nodes and gradually increased to deeper networks with more nodes per layers. We selected this approach since (1), has shown that random grid search outperforms normal grid search and manual search, and to make the results between models more comparable since they went through the same optimization pipeline.

Values in bold were the ones selected during grid search. Some chosen values, like the number of trees in a Random Forest (RFC), are in the extreme of the range. For the number of trees, since the Random Forest is an ensemble classifier, the more trees the higher the accuracy. However, the benefit becomes smaller as the number of trees grows, while the computation time continuously increases. The chosen number of trees is already quite extreme, and the gain from adding more trees is minimum, especially because many of the trees will be quite similar given the limited number of samples available. For tree depth in the Gradient boosting (XGB), the deeper the tree, the higher the risk of overfitting, therefore we set a maximum of 10 to prevent overfitting. (2,3)

Supplementary Table II. Hyper-parameters used for optimizing the Machine Learning models using grid-search. Values in bold indicate hyper-parameters chosen by the best model.

| **Classifier** | **Parameter Name** | **Parameter Value** |
| --- | --- | --- |
| **RFC** | Number of Trees | [100,200,400,600,800,1000,1200,**1400**] |
|  | Max features for split | **auto**, sqrt and log2 |
|  | Max depth of trees | [10,20,30,40, 50, 60, **70**, 80, 90, 100, None] |
|  | Quality of split | **Gini** or Entropy |
|  | Minimum number of samples required to split an internal node | [2,**4**,6,8] |
|  | Minimum number of samples required to be at a leaf node | [**2**,4,6,8,10] |
| **SVM** | Kernel type | Linear, **Radial basis function**, Polynomial |
|  | Penalty parameter C | [0.001, 0.01, 0.1, 1, 10, **100**] |
|  | Kernel coefficient γ (gamma) | [1, **0.1**, 0.01, 0.001, 0.0001] |
|  | Degree of the Polynomial kernel | [1,2,3,4,5,**6**] |
| **LR** | Regularization | [0.001, **0.01,** 0.1, 1, 10, 100] |
|  | Optimization algorithm | [newton-cg, lbfgs, liblinear, sag, **saga**] |
| **NN** | Hidden Layer sizes | [90,180,90], **[90,120,90]**, [90,90], [90,180], [90], [180] |
|  | Activation | ReLU, **logistic** |
|  | Regularization parameter | [0.1, **0.01**, 0.001, 0.0001] |
|  | Batch size | [32, **64**, 128] |
|  | Learning rate | [0.01, **0.001**, 0.005] |
|  | Optimization algorithm | **Adam** |
| **XGB** | Learning rate | [**0.1**, 0.01**,** 0.001, 0.005] |
|  | Minimum sum of instance weight (hessian) needed in a child | [**1,** 5, 10] |
|  | Minimum loss reduction required to make a further partition on a leaf node of the tree | [0**, 0.5**, 1, 1.5, 2, 5] |
|  | Subsample ratio of the training instances | [**0.7**, 0.8, 0.9, 1.0] |
|  | Parameters for subsampling the columns | [**0.3**,0.4,0.5,0.6,0.7,0.8] |
|  | Maximum depth of a tree | [3, 5, **7**, 9, **10**] |

Supplementary Table III - Extra evaluation measures in the testing data for all poor outcome prediction models, trained to maximize the AUC. The average of 10 cross validation iterations is presented. RFC, random forest classifier; SVM, support vector machine; LR, logistic regression; XGB, gradient boosting; NN, neural networks. AUC, area under the curve; NPV, negative predictive value; PPV, positive predictive value.

| **Method** | **Balanced Accuracy** | **MCC** |
| --- | --- | --- |
| RFC | 0.70 (0.68-0.72) | 0.41 (0.37-0.45) |
| SVM | 0.72 (0.70-0.74) | 0.42 (0.38-0.46) |
| NN | 0.71 (0.69-0.73) | 0.45 (0.41-0.50) |
| XGB | 0.71 (0.69-0.73) | 0.42 (0.37-0.46) |
| LR | 0.73 (0.71-0.75) | 0.44 (0.40-0.48) |

Supplementary Table IV - Evaluation measures in the testing data for all poor outcome prediction models, with the probability threshold optimized to 95% specificity. The average of 10 cross validation iterations is presented. RFC, random forest classifier; SVM, support vector machine; LR, logistic regression; XGB, gradient boosting; NN, neural networks. AUC, area under the curve; NPV, negative predictive value; PPV, positive predictive value.

| **Method** | **Specificity** | **Sensitivity** | **PPV** | **NPV** | **Balanced Accuracy** | **MCC** | **AUPRC** |
| --- | --- | --- | --- | --- | --- | --- | --- |
| RFC | 0.95 (0.93-0.96) | 0.31 (0.23-0.39) | 0.71 (0.63-0.79) | 0.74 (0.71-0.78) | 0.63 (0.59-0.67) | 0.34 (0.25-0.42) | 0.62 (0.56-0.68) |
| SVM | 0.93 (0.89-0.97) | 0.27 (0.20-0.42) | 0.63 (0.57-0.66) | 0.73 (0.69-0.77) | 0.56 (0.51-0.61) | 0.16 (0.07 -0.26) | 0.61 (0.53 -0.69) |
| NN | 0.95 (0.94-0.97) | 0.34 (0.29-0.39) | 0.77 (0.70-0.84) | 0.77 (0.70-0.84) | 0.65 (0.62-0.67) | 0.39 (0.34-0.44) | 0.66 (0.62-0.70) |
| XGB | 0.97 (0.95-0.99) | 0.21 (0.16-0.26) | 0.79 (0.71-0.87) | 0.79 (0.71-0.87) | 0.59 (0.58-0.61) | 0.30 (0.27-0.33) | 0.63 (0.59-0.66) |
| LR | 0.96 (0.94-0.97) | 0.31 (0.26-0.37) | 0.78 (0.71-0.86) | 0.78 (0.71-0.86) | 0.63 (0.61-0.66) | 0.38 (0.33-0.42) | 0.66 (0.62-0.70) |

Supplementary Table V - Evaluation measures in the testing data for all poor outcome prediction models, using the RFI and MICE imputation approaches. The average of 10 cross validation iterations is presented. RFC, random forest classifier; SVM, support vector machine; LR, logistic regression; XGB, gradient boosting; NN, neural networks. AUC, area under the curve; NPV, negative predictive value; PPV, positive predictive value, RFI: random forest imputation, MICE: multiple imputation by chained equations.

| **Method** | **AUC** | **Specificity** | **Sensitivity** | **PPV** | **NPV** | **MCC** | **AUPRC** |
| --- | --- | --- | --- | --- | --- | --- | --- |
| RFC - RFI | 0.80 (0.77-0.82) | 0.84 (0.81-0.86) | 0.56 (0.51-0.62) | 0.62 (0.56-0.68) | 0.80 (0.78-0.83) | 0.41 (0.37-0.45) | 0.66 (0.61-0.72) |
| RFC - MICE | 0.80 (0.77-0.82) | 0.81 (0.80-0.83) | 0.64 (0.61-0.68) | 0.63 (0.59-0.67) | 0.82 (0.80-0.84) | 0.41 (0.37-0.45) | 0.67 (0.63-0.72) |
| SVM- RFI | 0.77 (0.74-0.76) | 0.67 (0.61-0.72) | 0.78 (0.75-0.81) | 0.53 (0.48-0.57) | 0.87 (0.84-0.89) | 0.42 (0.38-0.46) | 0.69 (0.65-0.74) |
| SVM - MICE | 0.78 (0.76-0.80) | 0.67 (0.59-0.75) | 0.77 (0.73-0.81) | 0.55 (0.48-0.60) | 0.85 (0.83-0.87) | 0.42 (0.37-0.47) | 0.69 (0.65-0.73) |
| NN - RFI | 0.81 (0.79-0.83) | 0.89 (0.87-0.90) | 0.53 (0.49-0.57) | 0.69 (0.65-0.74) | 0.80 (0.78-0.83) | 0.45 (0.41-0.50) | 0.68 (0.64-0.73) |
| NN - MICE | 0.81 (0.78-0.83) | 0.88 (0.85-0.90) | 0.55 (0.50-0.60) | 0.69 (0.64-0.74) | 0.80 (0.77-0.83) | 0.46 (0.41-0.50) | 0.68 (0.64-0.72) |
| XGB- RFI | 0.78 (0.76-0.81) | 0.79 (0.76-0.83) | 0.63 (0.60-0.67) | 0.59 (0.54-0.65) | 0.82 (0.80-0.84) | 0.42 (0.37-0.46) | 0.64 (0.59-0.69) |
| XGB - MICE | 0.79 (0.77-0.81) | 0.75 (0.73-0.77) | 0.69 (0.65-0.73) | 0.58 (0.54-0.62) | 0.83 (0.81-0.85) | 0.43 (0.39-0.46) | 0.65 (0.61-0.69) |
| LR - RFI | 0.80 (0.78-0.82) | 0.75 (0.73-0.78) | 0.71 (0.68-0.73) | 0.57 (0.53-0.62) | 0.85 (0.83-0.86) | 0.44 (0.40-0.48) | 0.68 (0.63-0.74) |
| LR - MICE | 0.80 (0.78-0.82) | 0.73 (0.70-0.76) | 0.72 (0.69-0.76) | 0.57 (0.54-0.60) | 0.84 (0.82-0.87) | 0.43 (0.39-0.48) | 0.68 (0.63-0.72) |


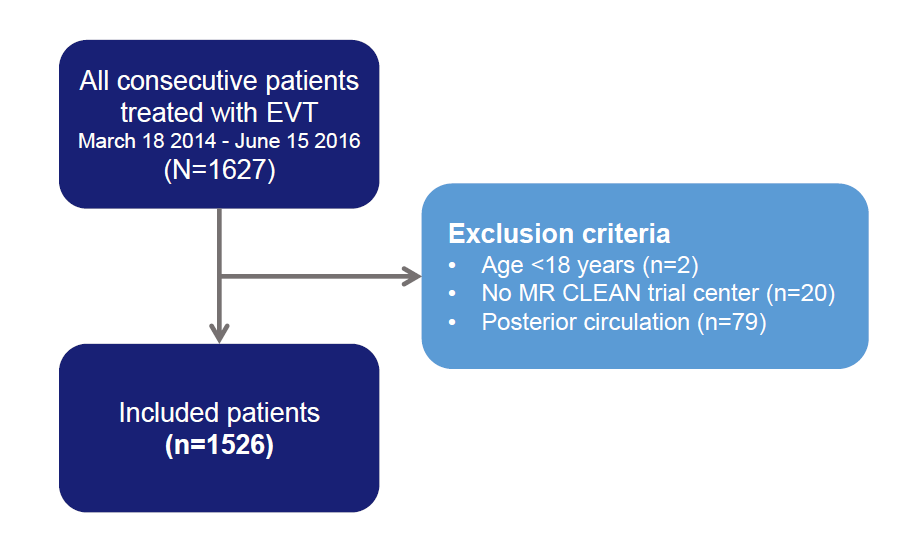


**Supplementary Figure I. Patient inclusion flowchart.** N denotes number of patients. EVT, endovascular treatment; MR CLEAN, multicenter randomized clinical trial for endovascular treatment of acute ischemic stroke in the Netherlands.

**
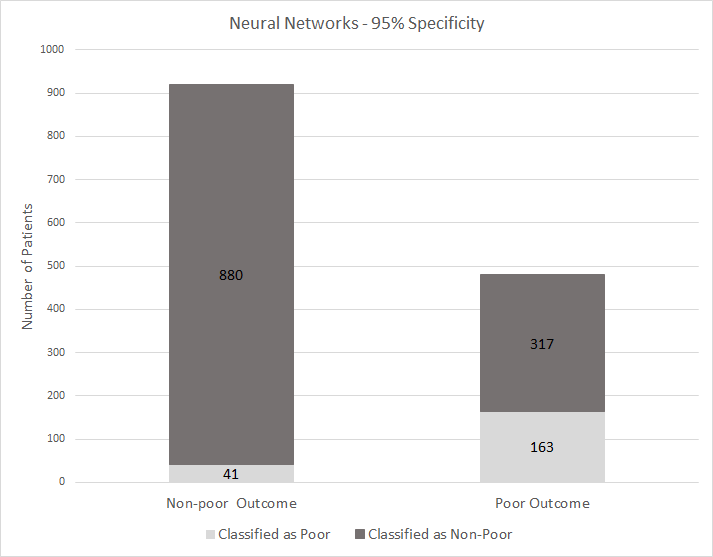
**

**Supplementary Figure II. Performance of poor outcome prediction neural network model trained for 95% specificity, in validation data.** Numbers in bars represent absolute number of patients.


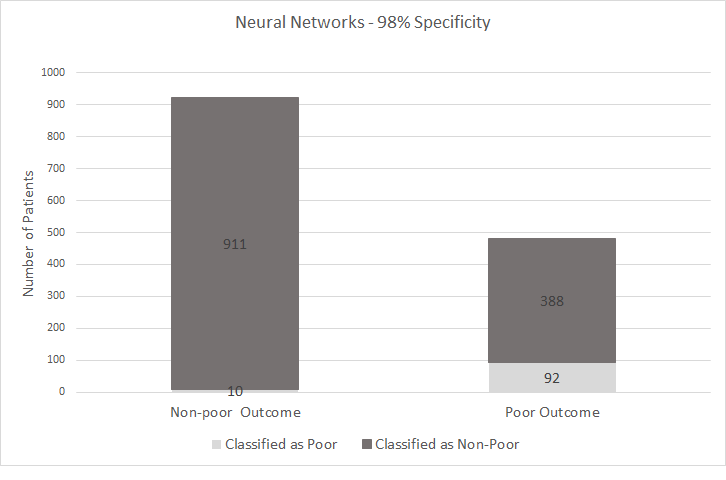


**Supplementary Figure III. Performance of poor outcome prediction neural network model trained for 98% specificity, in validation data.** Numbers in bars represent absolute number of patients.

*
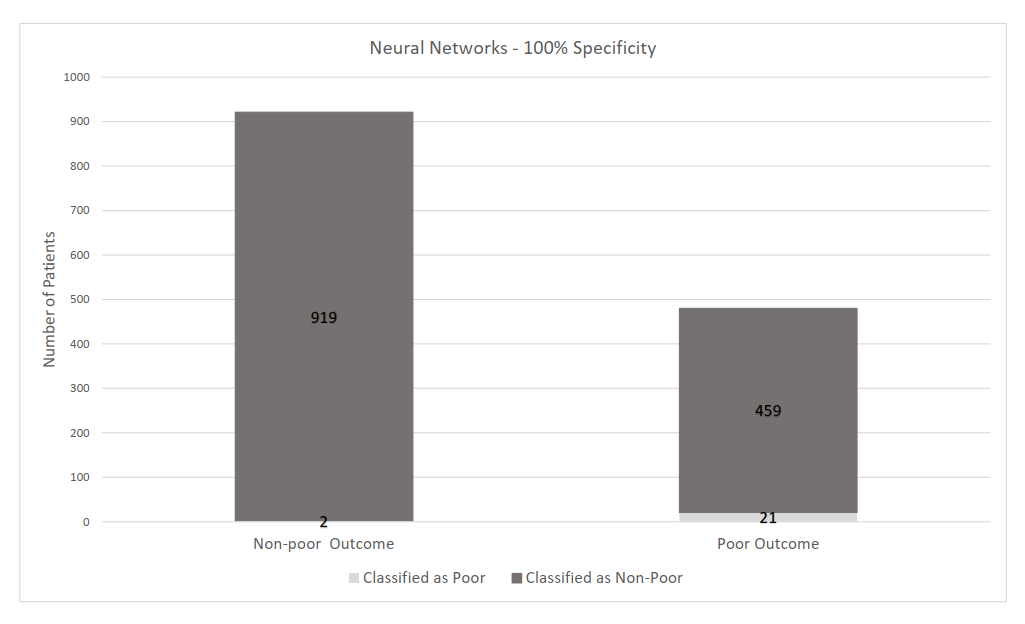
*

**Supplementary Figure IV. Performance of poor outcome prediction neural network model trained for 100% specificity, in validation data.** Numbers in bars represent absolute number of patients.


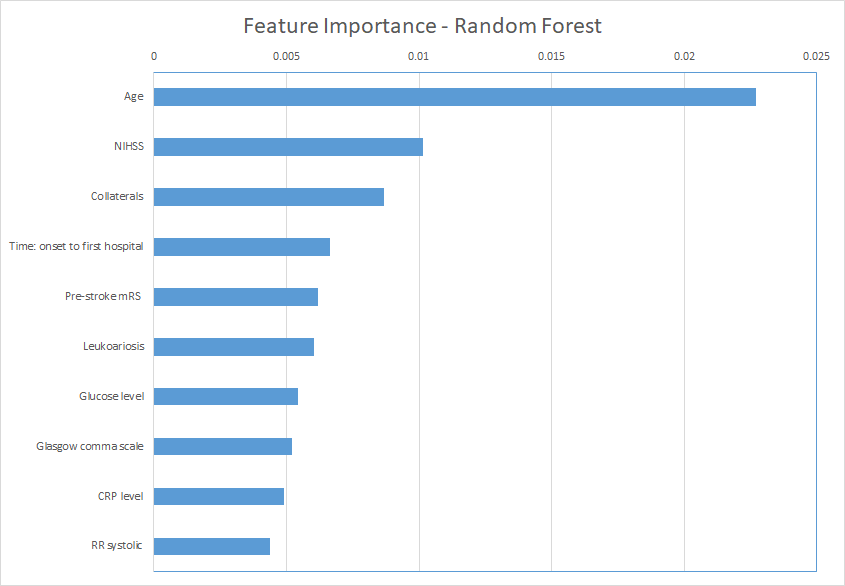


**Supplementary Figure VII. Permutation feature importance for the Random Forest models.** Average impact on the AUC. CRP, C-reactive protein; mRS, modified Rankin Scale; NIHSS, National Institutes of Health stroke scale; RR, blood pressure (Riva-Rocci).


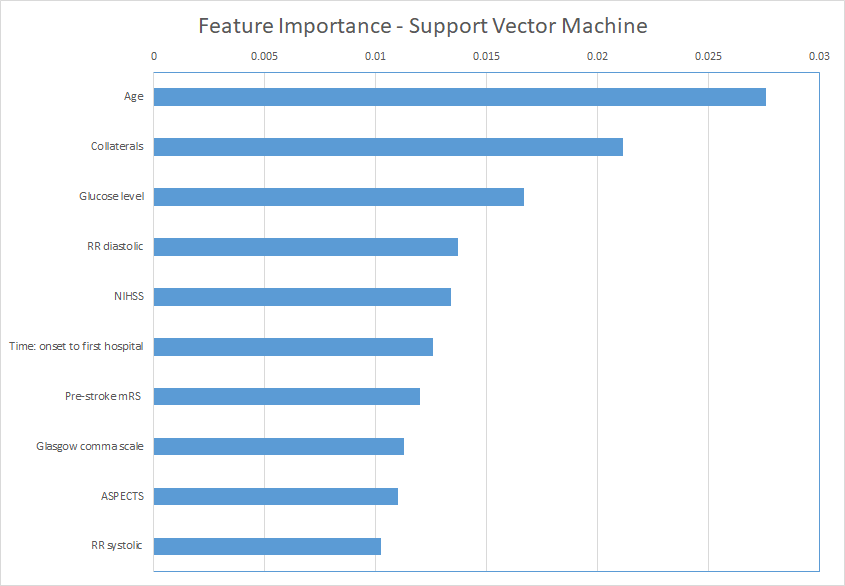


**Supplementary Figure VIII. Permutation feature importance for the Support Vector Machine models.** Average impact on the AUC. ASPECTS, Alberta Stroke Programme Early CT Score; CRP, C-reactive protein; mRS, modified Rankin Scale; NIHSS, National Institutes of Health stroke scale; RR, blood pressure (Riva-Rocci).


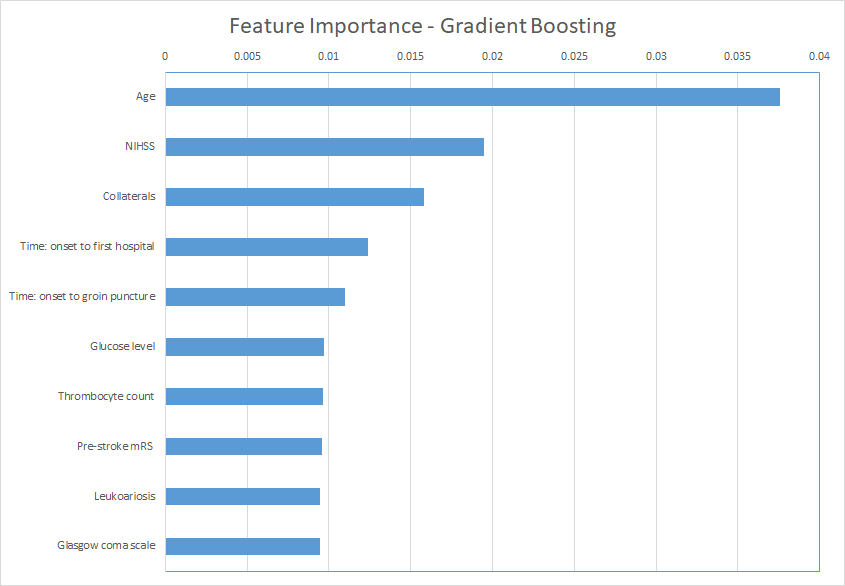


**Supplementary Figure IX. Permutation feature importance for the Gradient Boosting models.** Average impact on the AUC. mRS, modified Rankin Scale; NIHSS, National Institutes of Health stroke scale.

**References**

1. Bergstra JAMESBERGSTRA J, Yoshua Bengio YOSHUABENGIO U. Random Search for HyperParameter Optimization. J Mach Learn Res. 2012;13:281305.

2. Pedregosa F, Varoquaux G, Gramfort A, Michel V, Thirion B, Grisel O, et al. Scikit-learn: Machine Learning in Python. J Mach Learn Res. 2012;12:2825–30.

3. Chen T, Guestrin C. XGBoost: A Scalable Tree Boosting System. Proc 22nd ACM SIGKDD Int Conf Knowl Discov Data Min. 2016;Pages 785-794.
